# The Role of Orthophonia in Maxillo-facial Restoration

**Published:** 1919

**Authors:** 


**The Role of Orthophonia in Maxillo-facial Restoration.** By M. AD. BELANGER. *La Restauration Maxillo-faciale*, June, 1918.

The author insists upon the use of orthophonic processes following surgical facial restoration, and the necessity of resorting to orthophonia as early as possible. The alterations of pronunciation, due either to bad habits contracted involuntarily, to momentary physical defects, or to some ill-formation of lips, tongue, palate, teeth, nose, etc., can generally be greatly amended by appropriate exercises.

Except in cases of stammering, those whose speech is deficient are not aware of their faulty pronunciation. Their ear does not guide them, since it is incapable of discerning a bad pronunciation.

Orthophonic exercises prove most useful, and their efficiency has often been demonstrated in cases of severe war-wounds of the face.

The great thing is to resort to orthophonic reeducation as soon as possible, since it is as difficult to correct bad habits in pronunciation as in anything else.

